# Unlicensed medicines use: a UK guideline analysis using AGREE II

**DOI:** 10.1111/ijpp.12436

**Published:** 2018-01-30

**Authors:** Gemma Donovan, Lindsay Parkin, Lyn Brierley‐Jones, Scott Wilkes

**Affiliations:** ^1^ School of Pharmacy Pharmaceutical and Cosmetic Sciences Faculty of Health and Wellbeing University of Sunderland Sunderland UK; ^2^ Medicines Optimisation Team NHS Sunderland Clinical Commissioning Group Sunderland UK; ^3^ Pharmacy Department City Hospitals Sunderland NHS Foundation Trust Sunderland UK; ^4^ Department of Sociology Wentworth College University of York York UK; ^5^ 49 Marine Avenue Northumbria Primary Care Amble UK

**Keywords:** patient safety, evidence based practice, guidelines, interface issues, interprofessional issues

## Abstract

**Objectives:**

There is widespread use of unlicensed medicines within primary and secondary care but little information is available around how these medicines are used. This analysis examines and evaluates the content and quality of relevant guidance documentation currently in use within the UK.

**Methods:**

Guidance documents were identified through a literature search as well as email requests to pharmacy organisations throughout the UK. Unlicensed medicine documentation suitable for inclusion in the analysis underwent thematic analysis and quality assessment using the AGREE II tool.

**Key findings:**

Thematic analysis of 52 guidelines revealed four parent themes: (1) Professional responsibility (2) Usage practicalities (3) Risk versus benefit (4) Controlling use. There was variability in scores across the AGREE II domains with areas covering Scope and Purpose and Clarity of Presentation scoring well. Conversely, an area needing attention was Rigour of Development.

**Conclusion:**

Healthcare organisations would benefit from agreeing a ‘core content’ for the development of unlicensed medicines guidelines to ensure consistency and the presence of robust operating systems to deliver safe, effective treatment to all NHS patients.

## Introduction

An unlicensed medicine (ULM) is a medicinal product which has not been approved by a relevant regulatory body such as the Medicines and Healthcare Products Regulatory Agency (MHRA) in the UK. The term ‘unlicensed medicine’ incorporates a number of different categories of product which within the UK includes medicines manufactured in an MHRA licensed facility, medicines manufactured on a pharmacy premises under the supervision of a pharmacist and medicines imported from abroad without adoption of their native medicinal license by the MHRA. Within each of these categories, there is a varying range of safety and quality checks which are made prior to supply of the product. As with other medicines regulators, the legislation within the UK allows the use of unlicensed medicines for circumstances when existing licensed products are unavailable, unsuitable or are no longer able to meet the clinical needs of the patient. However, as there is no pharmaceutical license holder for an unlicensed medicine, the legal liability for their use rests between the prescriber and the dispensing pharmacist. Whilst some manufacturers will take some responsibility for the quality of the product, any adverse reactions relating to the medicine would be accountable to the prescriber who initiated the medicine and the pharmacist who dispensed it.

In the UK, an industry has been created around the manufacture of unlicensed medicines^1^ and there is also widespread manufacture within the National Health Service (NHS) in hospital manufacturing laboratories.^2^ Prescribing data from primary care within the NHS reveals that their use is widespread and that unlicensed products cause a significant financial burden on the health system.^3^ In the 12‐month period to September 2016, there was over £75 m spent on unlicensed medicines dispensed in community pharmacies in England.^4^

There is limited detailed guidance for the prescribing and dispensing of ULMs. Overarching principles have been set out by the MHRA;^5^ however, the main recommendation is that a licensed medicinal product should be used in preference to an unlicensed medicine. Use of an ULM from any category of source will have its own considerations depending on individual patient need and the extent to which quality and safety wish to be assured. Therefore, this high level guidance does not facilitate prescribers and pharmacists to make informed patient‐level decisions about the use of unlicensed medicines. All medicines regulators highlight that ULMs have not undergone the rigorous safety checks required to obtain approval.^5^, ^6^, ^7^, ^8^

Internationally, the European Medicines Agency (EMA) allows member states to use ‘unauthorised medicines’ in the same way as the UK. The Food and Drug Administration (FDA) in the United States refers to the use of unlicensed medicines as ‘Pharmacy Compounding’ and the Therapeutic Goods Administration (TGA) in Australia has a defined process for their use of ‘unapproved therapeutic goods’. Additionally in the UK, unlicensed medicines are also referred to as ‘Specials’, referring to the manufacturing facilities that make ULMs also being known as ‘Specials laboratories’. For the purposes of this article, we will use the term ‘unlicensed medicine’ to incorporate all of these terms. For clarity, the terms ‘off‐label’ and ‘unlicensed use’ describe using a licensed medicinal product outside of its approved particulars. For the purpose of this study, we focused on guidelines which were aimed at the use of unlicensed medicines. However, many of these documents also incorporated information around ‘off‐label’ and ‘unlicensed use’ of medicines, and so references to these were also incorporated where these were mentioned in relation to using unlicensed medicines.

### Aims and objectives

The aim of the guideline analysis was to:

- Assess the quality of guidelines developed to support use of ULMs within primary and secondary/tertiary care using an appropriate validated tool (AGREE II)
- Conduct a thematic analysis of the content of these guidelines
- To use these two data analysis techniques to identify areas of good agreement and areas for concern

## Methods

### Guideline document collection

Guidelines were sought nationally for inclusion in the analysis. This was performed through email requests, website and database searches. Email requests for unlicensed medicines guidelines were sent out locally and nationally through networks including local pharmaceutical committees, medicines management service providers, the Association of English Chief Pharmacists Network, the Association of British Pharmaceutical Specials Manufacturers and the Company Chemists Association. Notices were also added to electronic discussion boards on the UK Clinical Pharmacy Association and the Royal Pharmaceutical Society networks. The request sought any existing documentation guiding the procurement, use, administration or dispensing of unlicensed medicines. NHS organisation websites including NHS Trusts, Clinical Commissioning Groups and Local Pharmaceutical Committee were also searched for published guidelines. Websites of health organisations within the North East and North Cumbria Local Clinical Research Network Area were also hand searched for published guidelines for inclusion.

**Figure 1:**
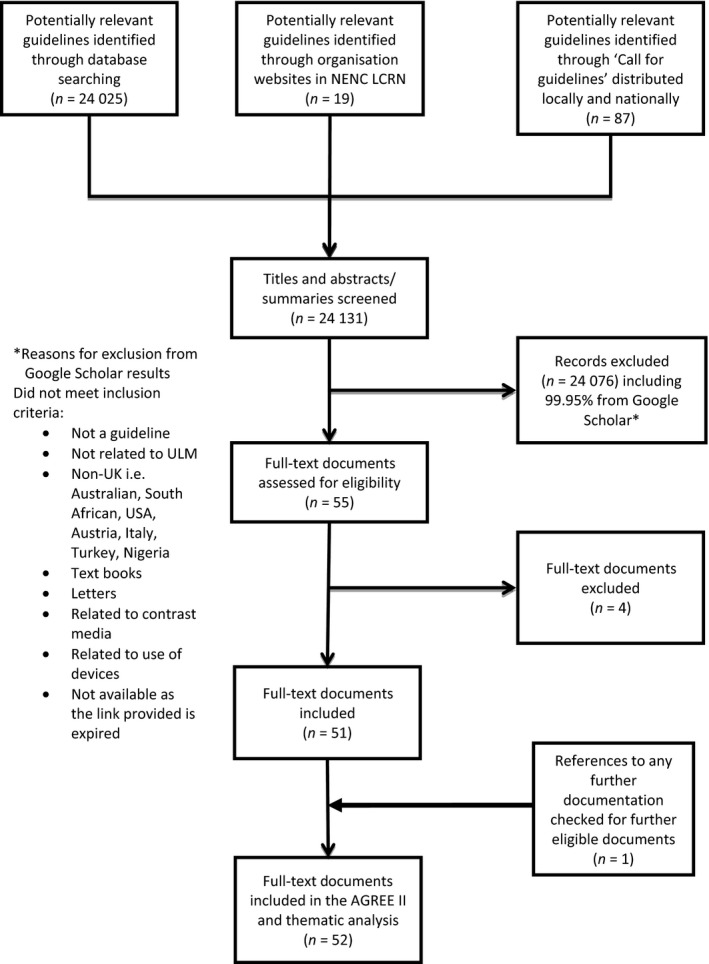
Flowchart for the identification of guidance documentation to be included in the analysis.

A search for guidelines from published literature was also performed between May 2015 and June 2015, searching the full‐text of articles using the search terms ‘unlicensed medicine’ OR ‘specials’ AND ‘policy’ OR ‘guideline’ OR ‘framework’ OR ‘standard operating procedure’ OR ‘standardised operating procedure’ OR ‘recommendation’ in Medline, International Pharmaceutical Abstracts, ISI Web of Knowledge, PubMed, Embase and Google Scholar (Figure 1).

Evaluation of the guidelines was carried out against inclusion and exclusion criteria (Table 1). Where guidelines covered more than the use of unlicensed medicines, for example prescribing policies, only the relevant sections were included in the analysis. For the purpose of this guideline analysis, we defined an unlicensed medicine as any medicinal product which does not possess a marketing authorisation from the MHRA (formerly known as a product license in the UK) and is intended for use as part of NHS treatment. We excluded guidelines which only dealt with off‐label use of medicines.

**Table 1:** Inclusion and exclusion criteria for guideline inclusion

Inclusion criteria	Exclusion criteria
Guidelines on the use of unlicensed medicines, including prescribing, procurement, dispensing or administration aimed for use within the UK NHS	Guidelines primarily relating to: Homeopathic medicines Food or dietary supplements Radiopharmacy Herbal medicines Off‐label or unlicensed use of licensed medicines Medical devices Investigational medicinal products Orphan drugs
Documents identified as guidelines, policies, frameworks, standard operating procedures or providing recommendations to inform use (as described above) of ULMs	Educational materials Stand‐alone patient information leaflets Formularies Newsletters Generalised guidance documents for prescribing or those relating to specific medicines or therapeutic areas Research studies on unlicensed medicines use
Any setting in which unlicensed medicines are used	Manufacturing guidelines

### Appraisal of guidelines for research and evaluation instrument II (AGREE II)

The AGREE II tool was used to evaluate the quality of the guidelines. AGREE II is an internationally recognised, validated tool^9^ used to assess both the quality and rigour of practice guidance by generating an overall guideline score.^10^ It utilises 23 pre‐validated items scored between 1 (strongly disagree) and 7 (strongly agree), organised into 6 domains:

- Scope and Purpose
- Clarity of Presentation
- Rigour of Development
- Editorial Independence
- Stakeholder Involvement
- Applicability

Each of these domains was scored for each of the unlicensed medicine guidelines included in the analysis. Scope and purpose is the domain which scores the extent to which the guideline is clear around why the ULM guideline is required and what elements of unlicensed medicines use will be included within it, for example prescribing, procurement, dispensing. Clarity of presentation allows scoring around whether it is clear what is expected from those using the guideline in terms of actions around ULM use. Rigour of development evaluates the use of evidence in formulating recommendations presented in ULM guidelines, for example clinical trials for recommending specific products. Editorial independence ascertains whether those who have been involved in writing the guideline are likely to have made decisions in an objective way and whether there may be conflicts of interest. This includes considerations such as whether an author stands to profit from use of a particular supplier or product which may be contained within the guideline. Stakeholder involvement ascertains if the right people have been involved in the process of developing the guideline, in the case of unlicensed medicines have the view of patients, doctors, dentists, nurses, pharmacists been included. And finally the applicability domain assesses whether the guideline authors considered how their recommendations can be best implemented and monitored through the provision of tools to facilitate guideline adoption, for example patient information leaflets and audit tools.

All 23 items were scored independently by two researchers (GD and LP). It is important to note that guideline ratings such as AGREE II do require a level of judgement. Both are pharmacists teaching within a UK university and have extensive experience in the NHS. GD has a background in primary care in community pharmacy, within GP practices and at a strategic level within a commissioning organisation. LP has a background in secondary care pharmacy practice and is an independent pharmacist prescriber. Both have had extensive experience of unlicensed medicines as part of their practice from a range of different viewpoints. Both completed online training on the use of the AGREE II tool.

As per guidance from AGREE, a score of 1 was used where the information required to score an item was absent. The scores within each of the 6 domains were then used to calculate the overall domain score for each document as well as providing an overall guideline score for all included documentation (http://www.agreetrust.org/). A score of more than 60% has previously been considered the threshold for what is considered good quality.^11^, ^12^, ^13^

### Thematic analysis

Thematic analysis was also conducted on the documentation, using constant comparison to compare between documents and emerging categories.^14^ To generate initial themes, GD and LP analysed eight of the 52 documents concomitantly to ensure codes were identified and labelled consistently. The remaining guideline documents were then thematically analysed independently by both researchers prior to gaining consensus on the final themes. NVivo 10, a qualitative data software package, was used to manage the large volume of data.

## Results

Fifty‐two guideline documents were included in the analysis with the documents originating from a range of settings (see Table 2) and taking various formats. The origin of the guideline documents is anonymised due to commercial sensitivity.

**Table 2:** Setting in which guidelines are intended for use

Guideline setting	N	%
NHS secondary and tertiary care trusts	29	56
Professional bodies and regulators	12	23
Primary Care	11	21

### AGREE II scoring

The AGREE II scoring results by domain and guideline setting can be found in Table 3. In five of the six AGREE II quality domains, guidelines from the primary care setting had the lowest scores. Guidelines from professional bodies had the highest scores in five of the six AGREE II domains. Guidelines from the NHS secondary and tertiary care setting generally had higher scores that those from the primary care setting and were close to those produced by professional bodies.

**Table 3:** Average scores (%) across the AGREE II domains and guideline setting

Setting	AGREE II domains					
	Scope and purpose (%)	Clarity of presentation (%)	Rigor of development (%)	Editorial independence (%)	Stakeholder involvement (%)	Applicability (%)
Primary care	57.9	69.4	7.1	1	20.2	17.8
NHS secondary and tertiary care	73.3	69.3	12.6	1	31.5	27.3
Professional bodies	74.6	72.2	15.4	7	35.2	21.4
Overall domain score	70.6	70.4	12.1	2.6	30	23.9

The scores revealed that the 52 documents performed well in both the Scope and Purpose (70.6%) and the Clarity of Presentation (70.4%) domains. The majority of the documents outlined their specific objectives clearly, their scope was well described and the target audience was easily identifiable; the presentation of information was good, with the provision of specific and unambiguous key recommendations.

In contrast, the Rigour of Development domain (12.1%) and the Editorial Independence domain (2.6%) scored very poorly. A lack of documented reference to a clear evidence base contributed to the poor score in Rigour of Development. Editorial Independence was unclear in most documents, lacking information on funding bodies or competing interests in the development group.

The Stakeholder Involvement score (30%) revealed that it was not always apparent in the guidelines if the development group included a diverse mix of healthcare professionals. The involvement of the target population (patients within the NHS) also scored poorly. There was little or no evidence of involvement of patients across all guidelines.

The applicability domain score was relatively low (23.9%). Whilst some documents provided advice and tools to aid implementation of their recommendations, many did not and the potential barriers and facilitators to implementation were often unacknowledged.

On an individual guideline level, none of the guidelines included scored above 60% across all five domains. Four guidelines did score more than 60% for Stakeholder Involvement, two of these were from NHS secondary and tertiary care settings and two were published by professional bodies. None of the guidelines included in the analysis scored above 60% for Rigour of Development, Editorial Independence or Applicability.

### Thematic analysis

Thematic analysis revealed four parent themes across the documentation: accepting professional responsibility; the practicalities of using an ULM; risk versus benefit analysis; and controlling access to the use of ULMs. Within these themes, several sub‐themes were contained which are summarised in Table 4 and discussed below.

**Table 4:** Themes from thematic analysis of guidelines content

Parent theme	Sub‐themes
Professional responsibility	Understanding the definitions around unlicensed medicines Usage awareness of patients and professionals Individual and organisational responsibilities References to the guidance and legislation
Usage practicalities	Selecting the pharmaceutical formulation Role of the pharmacist and the wider pharmacy team in use management Patient involvement Stages of use Continuing treatment
Risk versus benefit	Evidence to support use Place in the treatment of a patient and potential alternatives Describing and assessing risk Reporting of errors and adverse effects
Controlling use	Costs Audit of use Restricting use Organisational decision making

### Professional responsibility

Consistent references were made to the need for awareness of the unlicensed status of the medicines, and there were a range of definitions (Table 5) in the documentation, although there was consensus that an unlicensed medicine was a product without a marketing authorisation.

**Table 5:** Different definitions used to describe unlicensed medicines

Specials Medicines prepared by a UK manufacturer but without a UK product license Investigational medicinal products Medicines withdrawn from the UK market Medicines obtained from a manufacturer with an MHRA specials license Off‐label medicines Extemporaneously prepared medicines Re‐packed medicines Chemicals used to treat rare metabolic disorders Individually prepared medicines Imported medicines Homeopathic medicines Intermediate products Reconstituted medicines Temporarily authorised medicines Near‐label use of medicines Manipulation of medicines prior to administration CE marked products Food supplements Use of medicines at variance to their licence Compounding Mixing of medicines Compassionate medicine use

There was a focus in the guidelines on prescribers’ responsibility to be aware that the medicine being initiated was unlicensed. There were also references to pharmacy staff, nurses, patients, carers and those commissioning NHS services to also be aware. It was sometimes highlighted that prescribers may be unaware of the unlicensed status of a medicine and some documents provided guidance on status identification. Awareness was not just linked to licensing status, but also included understanding the potential implications in terms of prescribing, administration, procurement and supply. A wide range of professional bodies and legislation highlighting the responsibilities of individuals and organisations was referenced. The terminology used in the documentation varied between responsibility, accountability and liability.

### Usage practicalities

Most documentation dealt with the practicalities of using ULMs well. The chronological stages represented in the process of use included: (1) prescribing; (2) procurement; (3) storage; (4) dispensing and (5) administration. Some documents also described quarantining unlicensed medicines following procurement but prior to dispensing. Clinical review of the patient for the ongoing appropriateness of use was also a commonly occurring theme. Many guidelines contained associated paperwork in relation to handling/use. This was well described and included the following: information about manufacturers; the need for documented records of discussions with patients; written request orders and capturing justifications for the use of the unlicensed medicine.

Procurement processes were described in detail, including considerations around consistency of product, supplier identities and timeliness of supply. Information was available in many of the documents to support selection of a pharmaceutical formulation, including advice on availability of different formulations and the choice of formulation components used. The need for assurance of product quality was a strongly emerging theme, with frequent reference to the need for sourcing from ‘reputable’ suppliers. However, many guidelines did not provide a list of reputable suppliers or discuss how to recognise manufacturers as being reputable. This was reflected during the AGREE II scoring within the applicability domain where the average score for the item ‘the guideline provides advice and/or tools on how the recommendations can be put into practice’ was 3.9.

Pharmacists and the wider pharmacy team were referred to in managing use. Pharmacists were described in the ‘checking’ of prescriptions, both in a clinical screening process for appropriateness and in accuracy checking of the dispensed product. The need for pharmacists to communicate effectively with prescribers and other healthcare professionals on the unlicensed status of the product and its appropriate use was frequently included. Pharmacists were also portrayed as sources of information and advice to professionals and patients.

Many documents discussed the need for informed patient consent prior to using an unlicensed medicine and the provision of information in leaflets and/or verbal communication.

The theme ‘continuing treatment’ was mainly in relation to the transfer of prescribing from secondary or tertiary care to primary care. A small number of documents also referenced the need for communication with the community pharmacy responsible for continuation of supply following transfer of care.

### Risk versus benefit

All guidelines covered some element of identifying, stratifying and/or mitigating risks. However, this was very variable and contributed in part to the low score of 12.1% in the domain of Rigour of Development. Within this domain, the item ‘the health benefits, side‐effects, and risks have been considered in formulating the recommendations’ had an average score of 2.6 which supports the variability observed in the thematic analysis. In establishing the benefit of using an ULM, there was reference made to the need for evidence to support the clinical decision making process – a small proportion made reference to the use of medicines information departments to aid in gathering evidence. More common was the use of informal methods to measure benefit, such as whether the patient's clinical condition seems to improve or whether the patient was more easily able to self‐administer the medication. This was also reflected in the AGREE II scoring within the item ‘the guideline presents monitoring and/or auditing criteria’ demonstrating an average score of 1.9 from all documents, highlighting few formal pathways of reviewing practice and updating policy. In a small number of cases, there was mention that there should be a requirement to publish findings (positive and/or negative) to support future use of ULMs by practitioners.

Attempts to place ULMs in the context of other potential treatment options were prevalent. Whilst there was strong agreement across the documentation that a licensed medicine should be used in preference to an unlicensed one, the guidelines differed in the scope of alternatives that were considered. A description of the alternatives that were described in the documentation can be found in Table 6. The preference order in which these were placed was inconsistent between guidelines. Another prevalent message was that in some instances, an unlicensed medicine was an appropriate option for a patient and reassurance that their use was common. This was particularly evident in the paediatric setting.

**Table 6:** Preferred options for unlicensed medicines described in UK guidelines

Unlicensed	Batch‐prepared preferred to individually prepared unlicensed medicines Unlicensed medicines which appear in the Drug Tariff Part VIIIB
Acceptance of other licensing	Registered medical devices Imported medicines which are licensed in their country of origin
Use outside of license	Manipulation of a licensed dosage form, such as crushing tablets or opening capsules Products licensed for veterinary purposes but not in humans
No medicine	Review of medicines for patients with polypharmacy to potentially discontinue a medicine which is no longer required, rather than continue a medicine which is unlicensed Use of self‐care advice rather than prescribing an unlicensed treatment where there is no licensed medicine available

Risks referred to the potential side effects and product preparation. The need to report any adverse drug reactions was reinforced, often with reference to the Yellow Card Scheme.^15^ Additional internal reporting was also required by some of guidelines, making reference to contacting pharmacy departments where unlicensed medicines were manufactured on‐site. Some also referred to the need to report product defects and medication errors.

There was overall agreement across documents that an unlicensed medicine generally posed a greater risk to patient safety than a licensed medicine. Some described strategies for the risk assessment of all unlicensed medicines to identify and minimise some of the associated risks. There were varying levels of detail; some incorporated risk assessment documentation, others included strategies for mitigating the identified risks. There is a list of considerations that were described in the documentation as part of risk assessment for unlicensed medicines in Table 7.

**Table 7:** Risk assessment considerations contained in guidelines for unlicensed medicines

Area	Examples/Details
Therapeutic considerations of the active pharmaceutical ingredient	Indication, dose, side effects, interactions
Anticipated duration of use	Short term course of treatment, use to fill a gap in supply of a licensed medicine
Availability of analytical information for the product	Certificates of conformity, certificates of analysis, results of regional quality assurance testing, results of in‐house testing
Availability of product information	List of active pharmaceutical ingredient and excipients along with quantities, indications, instructions for use
Country of origin	UK, countries with whom there is a reciprocal acknowledgement of quality of pharmaceutical manufacture
Evidence for use	Whether research evidence exists, or whether the suggested use is known locally or nationally
Supplier	Whether the supplier is also the manufacturer, or an intermediary, if the manufacturer is licensed or known to the organisation e.g. NHS Manufacturing units
Information for use	Whether the product is labelled in English, if there is a patient information leaflet available
Route of administration	Products to be used topically considered to be of lower risk, with parenteral products considered higher risk
Standard availability	Is there a standard for the manufacture of the product such as a Pharmacopoeia monograph
Whether the product is considered a ‘medicine’	Products manufactured not for medicinal purposes such as food supplements
Withholding treatment impact	Consideration of whether the risk of withholding a treatment also has associated risks

### Controlling use

Restricting use of ULMs was described in many cases by limiting product use to meet individual patient needs, for example allergies to excipients in licensed products.

A requirement for organisational approval was evident mainly within the secondary or tertiary care setting prior to the use of an ULM. Some professional bodies such as the British Association of Dermatologists^16^ have published a list of recommended ULMs and their associated uses.

Prescriber constraints usually related to seniority, limiting initiation to consultant grades rather than junior medical staff. Some guidelines indicated restricted prescribing to secondary or tertiary care, identifying some ULMs as unsuitable for transferring to primary care. Many documents described using audits to ensure that unlicensed medicines were being used in accordance with the restrictions laid out within the guidelines but overall this was not detailed. Many referred to general auditing against the guideline rather than specifying any particular methods, measures or outcomes to be used – this was also supported by the AGREE II score within the applicability domain.

Costs relating to ULMs were a common feature of the documentation. Some simply highlighted variability and the need for sourcing from cost‐effective suppliers. Others made specific recommendations stating the preferred suppliers and details of the costs. Some guidelines made reference to the preferred use of a product included in the Drug Tariff^17^ Part VIIIB, a standardised price list which has been created for the reimbursement for a select number of unlicensed medicines dispensed in primary care in England and Wales. A number also made reference to ensuring that a licensed product was used in preference to an unlicensed one even where the former may be more expensive.

## Discussion

The scores from the AGREE II scoring found wide variability in the quality of guidelines for unlicensed medicines. We have also found that there is a lack of consistency of content across UK ULM guidelines. Whilst there was some agreement in content across the unlicensed medicines guidelines, a lack of a consistent approach is a cause for concern.

Inconsistency in content between guidelines may in part be due to the lack of a clear evidence base on which the guidelines have been developed. Lack of an evidence base for using unlicensed medicines was consistent throughout and is evident in the poor Rigour of Development domain score. This low score has also been reflected in other AGREE II analyses of guidelines where evidence is lacking for treatment recommendations such as paediatric headache^18^ and sedation in palliative care.^19^ Low scores identified within this analysis for the Rigour of Development domain echo the findings in dermatology guideline analysis.^12^ For Editorial Independence, a lack of clarity across funding bodies for guideline development or competing interests in the development group has also been found in other guidelines analysed using the AGREE II tool.^12^, ^18^

In agreement with our findings, a review of dementia guidelines found that Scope and Purpose, and Clarity of Presentation were the only domains that had high quality scores.^20^ Our poor score for Stakeholder Involvement highlighted a lack of transparency in developing, writing and updating guidelines. Organisations who use ‘working party’ methodology, such as the National Institute of Health and Clinical Excellence^11^, ^13^ and the Scottish Intercollegiate Guideline Network,^11^, ^13^, ^20^ generally score well in this domain. The Stakeholder Involvement domain also identified a deficit in patient involvement in guideline development, similar to other findings using AGREE II.^11^, ^21^

This study also found variation in terminology in terms of what constitutes an ‘unlicensed medicine’ which is potentially misleading for both healthcare professionals and their patients. Lack of clarity in describing the roles and responsibilities of the individual members of the healthcare team also exists. This could make implementation of such guidelines difficult and is reflected in low scores in the ‘applicability’ domain. Other analyses have also found a wide variability in the consideration of the use of guidelines in practice.^11^, ^12^, ^18^, ^20^, ^21^

Risk attenuation procedures for the use of ULMs were heavily focused on pharmacy teams. In contrast, prescribers are seen to be responsible for the clinical decision making, documentation of communication with the patient and transferring use of unlicensed medicines across care settings. There was no guidance in the event that the primary care prescriber did not want to take over prescribing responsibility. This is a difficult area as it is especially important that prescribers in primary care are confident in taking on the transferred responsibility from secondary or tertiary care.^22^

It is reassuring to see that most of the guidelines made reference to involving patients in the decision to use a ULM. However, not all guidelines provided the tools to facilitate these conversations and within the paediatric setting the general public lacked knowledge about ULM use.^23^ A questionnaire study also found that there was little knowledge of the licensing process of medication^24^ leaving patients poorly equipped to facilitate informed decision making and to ensure safe and appropriate use of their medication.

The lack of consistency around the assessment of risks and benefits is probably the greatest area of concern revealed in this study. If there is not agreement about how risk in using ULMs should be assessed, then there is likely to be variability in the products which are ultimately chosen for procurement. Some tools may also be based on a larger body of intelligence gained from experience that may not be widely shared, so organisations without this intelligence may continue to use products that have been disregarded elsewhere due to safety concerns. Some organisations placed responsibility for the entire process on a single clinician, a strategy that potentially increases the risk for patients as that clinician may not have the knowledge and skills to perform a comprehensive risk assessment. Other guidelines recommended involvement of the multi‐disciplinary team, and it would be appropriate to adopt this approach more widely. The main concerns around the associated risks have also been documented by Chisolm^24^ who argued that safety is one of the most important aspects when prescribing ULMs alongside efficacy.

A phenomenological review of the ULM literature^25^ revealed that adverse events are unreported for ULMs. This is surprising given that within the guideline analysis conducted here, recommendations around the reporting of adverse drug reactions are evident. On the other hand, there is little information in the guidelines around ensuring that a licensed product is dispensed where the medication is prescribed generically, a point also highlighted by Sutherland and Waldeck.^25^ This may be due to this information being contained within dispensary‐based standard operating procedures not submitted as part of the call for guidelines in this study.

The General Medical Council (GMC) guideline^26^ states that ULMs may be prescribed where there is no suitable licensed alternative that can meet the patient's need. However, this analysis suggests that there is widespread use of ULMs to meet the needs of specific patient groups and for specific indications. These routine uses are commonly documented in local ULM guidelines and therefore are potentially seen to be legitimised (see also^25^).

Whilst the AGREE II tool is used to assess the quality of national guidelines such as that produced by NICE and the guidelines included here also incorporate local guidelines and procedures, the standards to which these are produced in terms of process would be expected to be the same. For unlicensed medicines, the absence of a robust national guideline means that local procedures are even more important in guiding local decision making. However, we also acknowledge that the resources available to develop guidelines at a local level will be significantly reduced compared to that available to national guidance bodies.

### Limitations to this study

Our analysis has given a clearer picture of the variability in content and quality of guidelines currently in use for ULMs within the UK. However, 56% of these documents came from secondary and tertiary care centres compared to 21% from primary care. It is unclear whether the smaller number from primary care is due to a lack of available guidelines or a lack of submission to the project. Although there were submissions to the analysis from across England, the submissions may not be representative of all guidelines within the UK. There were also no submissions from private healthcare organisations. Whilst all efforts were made to identify documents for inclusion, we acknowledge that there will be more documentation which could have been included. However, this would be almost impossible to collect at this large scale and analysis of all of those guidelines would be impracticable. It should be noted that other studies of this type have analysed much smaller samples of guideline documentation. We used two reviewers for the analysis which is within recommendations for using the AGREE II tool; however, more reviewers would increase the reliability of the scores. Whilst we also attempted to find information to rate guidelines using other sources that were submitted alongside guidelines or websites of the organisations who published the guidelines, we did not contact authors or organisations directly to source additional information where this was not readily available. This could mean that some scores could be lower due information not being available which may be available to users of the guideline.

Document analysis can also only provide part of the picture in relation to ULM use. Here, we have used guidelines to explore how organisations are recommending that ULMs are handled, and these may not be borne out in the actions of healthcare professionals. Further research is required to explore how guideline recommendations translate into clinical practice.

## Conclusion

When producing guidelines for ULM usage, organisations should pay attention to the rigour of their development, stakeholder involvement and editorial independence. In addition, our thematic analysis also identified the need to focus on professional responsibility, the practicalities of using a ULM, improved risk/benefit analysis and how access to the use of ULMs is controlled.

A ‘formulary’ of unlicensed medicines is a potential solution to the problem of ULM use. In turn, this may aid improved monitoring through existing pharmacovigilance infrastructure, a process that could also be supported with dosing information for prescribers, compilation of recommended alternatives and other therapeutic information such as cautions, contraindications and dose adjustments. It may also reduce some of the organisation‐level assessments that are made within the NHS and could release efficiency savings which has been a focus of the Carter Review.^27^ However, this approach may run contrary to the legislative basis on which ULMs are used, that they should only be used to meet the needs of an individual patient. An alternative would be a legislative approach similar to that in Australia whereby the use of unlicensed medicines is approved centrally either for individual prescribers or on a case‐by‐case basis.^7^

There is a need for national leadership in the UK to develop a strategy in relation to the use of unlicensed medicines. This should include clear guidance on risk assessment and their place in the clinical management of patients. These decisions should take into account the views of stakeholders from a range of expertise as well as patients. Only then can we be confident that when an ULM is used, all considerations have been taken into account to ensure it is the best treatment option in the absence of a licensed medicine.

## Declarations
